# DNA methylation signatures in Blood DNA of Hutchinson–Gilford Progeria syndrome

**DOI:** 10.1111/acel.13555

**Published:** 2022-01-19

**Authors:** Yosra Bejaoui, Aleem Razzaq, Noha A. Yousri, Junko Oshima, Andre Megarbane, Abeer Qannan, Ramya Potabattula, Tanvir Alam, George M. Martin, Henning F. Horn, Thomas Haaf, Steve Horvath, Nady El Hajj

**Affiliations:** ^1^ College of Health and Life Sciences Qatar Foundation Hamad Bin Khalifa University Doha Qatar; ^2^ Genetic Medicine Weill Cornell Medicine‐Qatar Doha Qatar; ^3^ Department of Laboratory Medicine and Pathology University of Washington Seattle Washington USA; ^4^ Department of Clinical Cell Biology and Medicine Graduate School of Medicine Chiba University Chiba Japan; ^5^ Department of Human Genetics Gilbert and Rose‐Marie Ghagoury School of Medicine Lebanese American University Byblos Lebanon; ^6^ Institut Jérôme Lejeune Paris France; ^7^ Institute of Human Genetics Julius Maximilians University Würzburg Germany; ^8^ College of Science and Engineering Hamad Bin Khalifa University Doha Qatar; ^9^ Department of Human Genetics David Geffen School of Medicine University of California Los Angeles Los Angeles California USA; ^10^ Department of Biostatistics Fielding School of Public Health University of California Los Angeles Los Angeles California USA

**Keywords:** accelerated aging, DNA methylation, epigenetic clock, Hutchinson–Gilford Progeria syndrome, progeroid laminopathies

## Abstract

Hutchinson–Gilford Progeria Syndrome (HGPS) is an extremely rare genetic disorder caused by mutations in the *LMNA* gene and characterized by premature and accelerated aging beginning in childhood. In this study, we performed the first genome‐wide methylation analysis on blood DNA of 15 patients with progeroid laminopathies using Infinium Methylation EPIC arrays including 8 patients with classical HGPS. We could observe DNA methylation alterations at 61 CpG sites as well as 32 significant regions following a 5 Kb tiling analysis. Differentially methylated probes were enriched for phosphatidylinositol biosynthetic process, phospholipid biosynthetic process, sarcoplasm, sarcoplasmic reticulum, phosphatase regulator activity, glycerolipid biosynthetic process, glycerophospholipid biosynthetic process, and phosphatidylinositol metabolic process. Differential methylation analysis at the level of promoters and CpG islands revealed no significant methylation changes in blood DNA of progeroid laminopathy patients. Nevertheless, we could observe significant methylation differences in classic HGPS when specifically looking at probes overlapping solo‐WCGW partially methylated domains. Comparing aberrantly methylated sites in progeroid laminopathies, classic Werner syndrome, and Down syndrome revealed a common significantly hypermethylated region in close vicinity to the transcription start site of a long non‐coding RNA located anti‐sense to the Catenin Beta Interacting Protein 1 gene (*CTNNBIP1*). By characterizing epigenetically altered sites, we identify possible pathways/mechanisms that might have a role in the accelerated aging of progeroid laminopathies.

## Graphical abstract

Hutchinson–Gilford Progeria Syndrome (HGPS) is characterized by premature and accelerated aging beginning in childhood. DNA methylation age acceleration was not observed in peripheral blood DNA of HGPS patients; however, a significant hypomethylation was detected in CpG sites overlapping solo‐WCGW partially methylated domains.

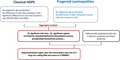

## 1 INTRODUCTION

The nuclear envelope is composed of a double lipid bilayer and an underlying network of intermediate filament proteins that make up the nuclear lamina. Principal components of the mammalian nuclear lamina are lamins A, B1, B2, and C (Recently reviewed in (Wong & Stewart, 2020)). Lamins A and C are splice isoforms from the same gene (*LMNA*), while lamins B1 and B2 are each coded for by separate genes. A number of diseases associated with mutations in nuclear lamins and lamin‐associated proteins have been collectively termed laminopathies(Burke & Stewart, 2006; Worman, 2012). The majority of laminopathies are due to variants found in the *LMNA* gene, which to date has over 600 variants reported (de Leeuw et al., 2018). Laminopathies are classified into some 30 diseases and conditions, which fall into three larger categories: lipodystrophies, muscular dystrophies, and premature aging (Wong & Stewart, 2020). Hutchinson–Gilford Progeria Syndrome (HGPS) is the most severe form of premature aging associated with variations in lamin A (De Sandre‐Giovannoli et al., 2003; Eriksson et al., 2003).

Lamin A protein undergoes a series of posttranslational processing steps that are important for its normal function. Briefly, prelamin A contains a C‐terminal CaaX motif, the cysteine of which is farnesylated by a farnesyl transferase. The three C‐terminal amino acids are then removed by either RAS‐converting enzyme 1 (RCE1) or ZMPSTE24 (FACE1) and the farnesylated cysteine is methylated. The final cleavage by ZMPSTE24 results in removal of 15 C‐terminal amino acids resulting in a mature lamin A (Davies et al., 2011; Sinensky et al., 1994). In HGPS, a C>T substitution at position 1824 creates a cryptic splice site in the lamin A mRNA. This results in the removal of 50 amino acids, which contain the second ZMPSTE24 cleavage site (De Sandre‐Giovannoli et al., 2003; Eriksson et al., 2003). This shorter form of lamin A, also known as progerin, is constitutively tagged at the C‐terminus by a farnesyl cysteine methyl ester. Individuals affected by HGPS experience short stature, bone loss, lipodystrophy, and alopecia, with most patients suffering from fatal heart failure in their early teens (Hennekam, 2006; Vidak & Foisner, 2016). The molecular disease progression is thought to involve at least two cellular aspects, the organization and maintenance of DNA, and the mechanical resilience of the nucleus.

Chromatin is organized into topological associated domains (TADs) (Dixon et al., 2012; Lieberman‐Aiden et al., 2009). Some TADs have been shown to interact with the nuclear envelope through Lamin‐Associated Domains (LADs), which are transcriptionally repressed regions (Lochs et al., 2019). As such, the nuclear lamina can regulate chromatin by promoting interaction with LADs, though the mechanisms by which the interaction of chromatin and the nuclear lamina is regulated remains an area of active research (Wong & Stewart, 2020). Even though the genetic mutations causing HGPS have been known for years, the molecular processes underlying the phenotype remain to be clarified. One mechanism for translating the effects of specific gene mutations into the associated comorbidities of premature aging is through epigenetic dysregulation of relevant genes/pathways. Several epigenetic alterations were reported to occur in HGPS cells including downregulation of H3K27me3 and H3K9me3 as well as upregulation of H4K20me3 (McCord et al., 2013; Shumaker et al., 2006). Moreover, HGPS cells were shown to display DNA methylation aberrations across several regions. A study by Liu et al. measured DNA methylation of 95,932 CpG sites in HGPS fibroblasts using targeted bisulfite padlock probes followed by sequencing (Liu et al., 2011). This revealed 586 genes containing HGPS differentially methylated regions that play a role in development and transcriptional regulation. On the contrary, induced‐pluripotent stem cells (iPSCs) from HGPS patients only showed DNA methylation abnormalities in 33 autosomal genes. A novel DNA methylation age clock based on 391 CpG sites also displayed epigenetic age acceleration in HGPS fibroblasts (Horvath et al., 2018). More recently, a comprehensive study by Köhler et al. analyzed chromatin accessibility via transposase‐accessible chromatin with ‐visualization/‐sequencing (ATAC‐see/‐seq) and measured DNA methylation using Illumina EPIC Methylation arrays in 9 primary fibroblasts of HGPS patients vs 6 control samples. This revealed the enrichment for chromatin accessibility changes and DNA methylation aberrations in LADs of HGPS patients (Kohler et al., 2020). A study by Heyn et al. has looked at differential DNA methylation of EBV‐transformed B cells in patients with Werner syndrome (WS) and in a family with progeroid features presenting a HGP‐like phenotype(Heyn et al., 2013). EBV immortalization is known to cause large‐scale hypomethylated blocks across the genome, and this is why the authors could only study DNA methylation in a subset of the measured CpG sites (272,290 out of 485,577), as several sites were filtered out because of inconsistent DNA methylation between naive and immortalized samples (Hansen et al., 2014). Until now, no study has investigated DNA methylation alterations in blood DNA of HGPS patients, which is inherently related to the very limited number of HGPS patients. To fill this gap, we have performed the first comprehensive genome‐wide DNA methylation analysis in peripheral blood DNA of 8 classic HGPS patients and 7 patients with non‐classical progeroid laminopathy including matched healthy controls.

## 2 RESULTS

### 2.1 DNA methylation alterations in progeroid laminopathies

We used the Infinium MethylationEPIC BeadChip to compare genome‐wide DNA methylation signatures in whole blood DNA of progeroid laminopathy patients with *LMNA* mutations versus age‐ and gender‐matched controls. Differentially methylated sites and regions (genes, promoters, CpG islands, and tiling regions) between samples were analyzed following adjustment for age and gender and cell type composition via the RefFreeEWAS package(Houseman et al., 2014). An initial differential methylation analysis comparing 8 classical HGPS vs age‐ and gender‐matched controls and 7 progeroid laminopathy patients (non‐classical mutation) vs matched controls revealed no differentially methylated sites/regions with a false discovery rate (FDR)‐adjusted *p* value < 0.05 in both comparisons. In order to increase sample number to detect small effect size, we performed an aggregate analysis combining all progeroid laminopathies (*N* = 15) versus matched controls (*N* = 12). At the site level, this analysis revealed 61 differentially methylated sites with a FDR‐adjusted *p* value < 0.05 and a β methylation difference of >0.02 or <−0.02 (2% methylation difference) (Table S1). At the region level analysis, we observed no significant gene, promoter, or CpG island, whereas the 5 Kb tiling analysis revealed 32 significant regions when comparing progeroid laminopathies vs controls (Table S2). Next, we tested Gene Ontology (GO) enrichment for the 61 significant CpGs using the methylglm function implemented in the methylGSA package that performs gene set analysis following adjustment for the number of CpG sites per gene(Ren & Kuan, 2019). This revealed significant enrichment for 8 GO terms including phosphatidylinositol biosynthetic process, phospholipid biosynthetic process, sarcoplasm, sarcoplasmic reticulum, phosphatase regulator activity, glycerolipid biosynthetic process, glycerophospholipid biosynthetic process, and phosphatidylinositol metabolic process (Table 1). We additionally used *eFORGE* to perform functional overlap analysis for chromatin‐signal enrichment across specific cells or tissues(Breeze et al., 2019). However, we did not observe differentially methylated probes (DMPs) to be enriched at DNase I hypersensitive sites (DHSs) (Figure S1), 15 chromatin states, and 5 histone marks from the consolidated Roadmap Epigenomics Consortium. To test for the effect of methylation alterations on the expression of nearby genes, we performed an expression quantitative trait methylation (eQTM) analysis for the 61 DMPs via the Biobank‐based Integrative Omics Study (BIOS)–QTL browser. This analysis showed no association between methylation at these sites and expression of nearby genes (Table S1).

**TABLE 1:** Gene ontology enrichment for the 61 significant CpGs in blood DNA of patients with progeroid laminopathies following adjustment for number of CpG sites per gene on the Infinium Epic arrays

ID	Description	Size	*p* Value	*p*‐adj
GO:0006661	Phosphatidylinositol biosynthetic process	172	5.33E‐05	0.00319049
GO:0008654	Phospholipid biosynthetic process	386	5.33E‐05	0.00319049
GO:0016528	Sarcoplasm	124	5.33E‐05	0.00319049
GO:0016529	Sarcoplasmic reticulum	110	5.33E‐05	0.00319049
GO:0019208	Phosphatase regulator activity	121	5.33E‐05	0.00319049
GO:0045017	Glycerolipid biosynthetic process	372	5.33E‐05	0.00319049
GO:0046474	Glycerophospholipid biosynthetic process	319	5.33E‐05	0.00319049
GO:0046488	Phosphatidylinositol metabolic process	285	5.33E‐05	0.00319049
GO:0005085	Guanyl‐nucleotide exchange factor activity	308	0.00881494	0.30159679

### 2.2 Differentially methylated sites in progeroid laminopathies

**FIGURE 1:**
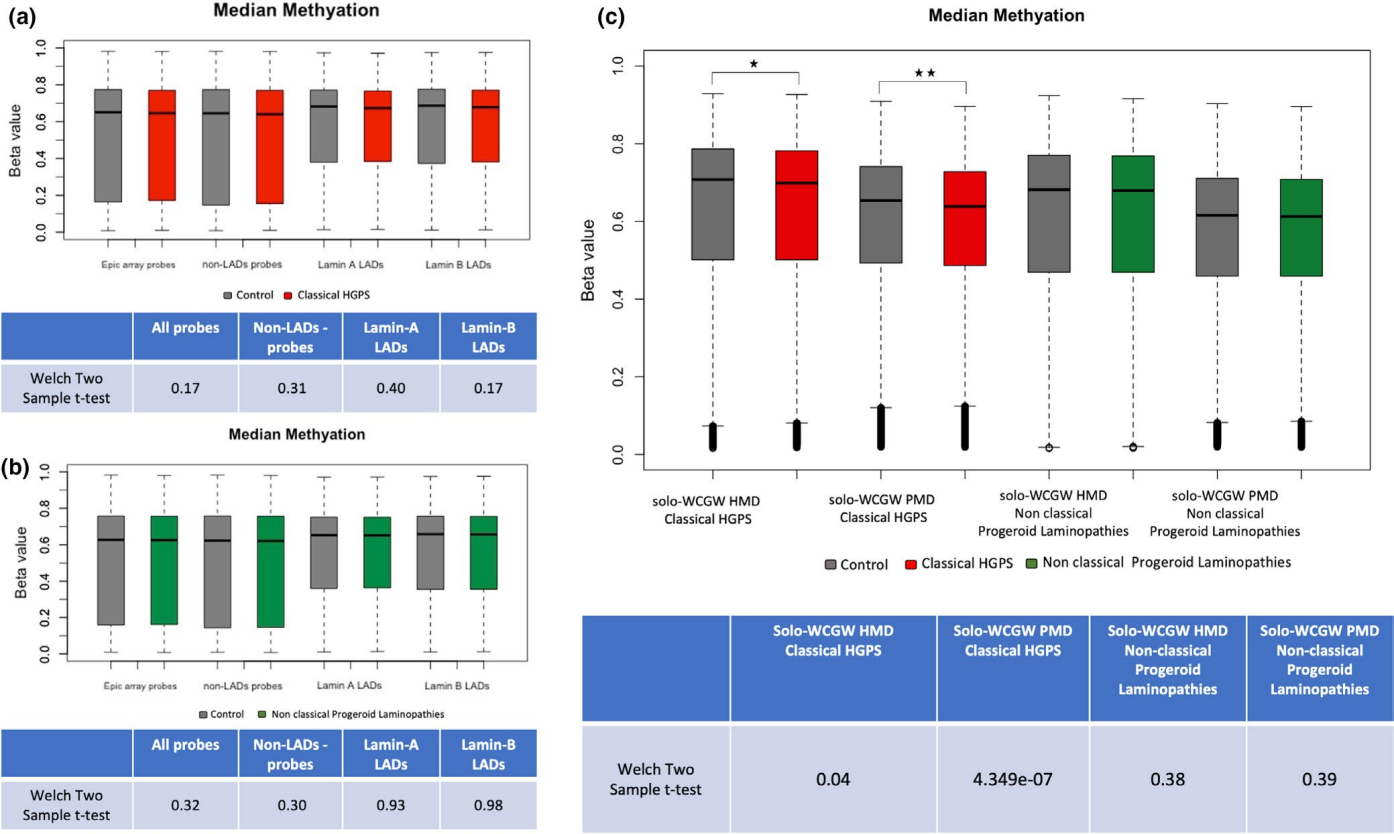
DNA methylation differences in (a) classical HGPS (red) (b) non‐classical progeroid laminopathies (green) vs controls (gray) for β values at probes located in Lamin A LADS and Lamin B LADs identified in HeLa cells. The Welch two‐sample t test was used to perform the statistical comparison between cases and controls. The median of β values is displayed as a solid black line **c**. DNA methylation levels of solo‐WCGW sites located in partially methylated (PMD) and highly methylated domains (HMD) in classical HGPS (red), non‐classical progeroid laminopathies (green), and the matched controls for each group (gray). Median is indicated by solid line

HGPS fibroblasts have been shown to have a loss of peripheral heterochromatin and associated H3K27me3 histone marks at the nuclear periphery(McCord et al., 2013). Therefore, we investigated whether CpG sites associated with genomic regions in contact with nuclear lamina are differentially methylated in blood DNA of HGPS patients. Here, we used a Welch two‐sample t test to compare methylation levels between classic HGPS and controls at probes located at lamin A LADs across several cells/tissues (Guelen et al., 2008; Lund et al., 2014; Lund et al., 2015; Meuleman et al., 2013). We observed no differences in DNA methylation across CpG sites residing in lamin A LADs identified in HELA cells (*p* value = 0.40) (Figure 1a), fibroblasts (*p* = 0.50), and the HT1080 cell line (*p* = 0.59). We additionally looked at redistributed LAD genomic regions in dilated cardiomyopathy (DCM) hearts with pathogenic variants in *LMNA* (Cheedipudi et al., 2019). Similarly, we did not observe difference in average methylation of CpG sites in those regions (*p* value = 0.17) when comparing classic HGPS patients vs controls. Next, we performed a similar analysis for patients with non‐classic progeroid laminopathies, which revealed no difference for CpG sites located in lamin A LADs of HeLa cells (*p* = 0.93) (Figure 1b), fibroblasts (*p* = 0.99), and the HT1080 cell line (*p* = 0.93) as well as regions exhibiting gains and losses of LADs in DCM patients (*p* = 0.81). Furthermore, we looked at methylation levels of solo‐WCGW sites located in partially methylated domains (PMD) and highly methylated domains (HMD) (Zhou et al., 2018). We observed significant methylation differences in solo‐WCGW PMDs (*p* value = 4.349e‐07) and HMDs (*p* = 0.04) when comparing classic HGPS patients vs controls, whereas in non‐classic progeroid laminopathies, probes overlapping solo‐WCGW PMDs and HMDs revealed no differences, *p* = 0.39 and *p* = 0.38, respectively (Figure 1c).

### 2.3 Epigenetic aging in Progeroid Laminopathies

**FIGURE 2:**
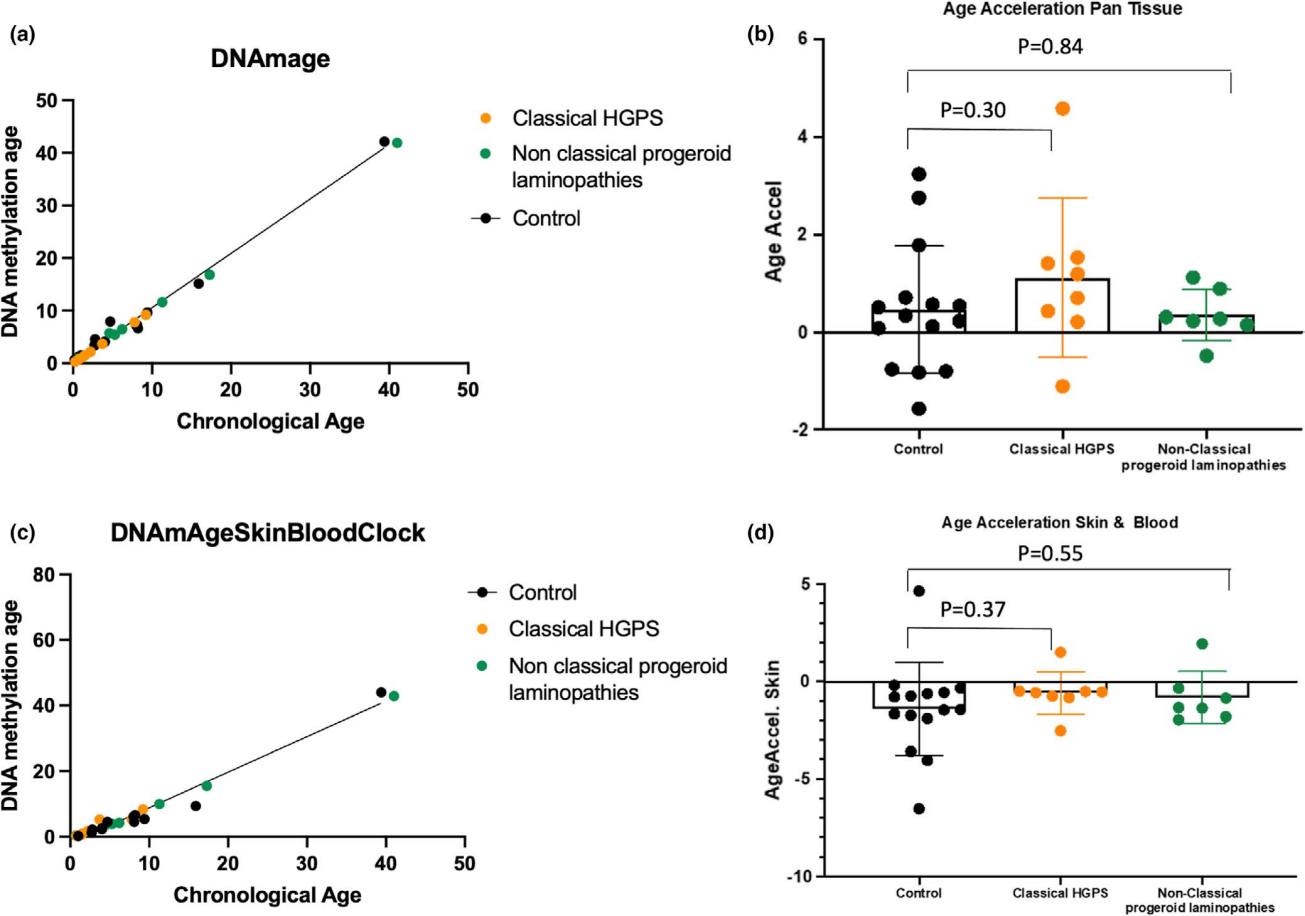
(a) Chronological age (x‐axis) vs DNA methylation age (y‐axis) and (b) age acceleration measured using the Horvath clock as well as (c–d) DNAmAgeSkinBloodClock

Epigenetic clocks were reported to show accelerated aging in progeroid syndromes including fibroblasts from HGPS syndrome. For this reason, we looked at epigenetic age in blood DNA of our samples. Most of the studied patients were <20 years old; therefore, we used the pan‐tissue Horvath clock and the skin and blood clock since these two clocks can be applied to blood samples from children. This analysis revealed that classic HGPS and non‐classic progeroid laminopathy patients are not associated with epigenetic age acceleration in blood (Figure 2). We also compared measured epigenetic age acceleration (EEAA) and intrinsic epigenetic age acceleration (IEAA). We could observe significant difference when comparing non‐classic progeroid laminopathies vs controls (*p* = 0.035), whereas classic HGPS showed no differences (*p* = 0.88) (Figure S2). We additionally performed an analysis focused on samples <10 years old across all groups, which similarly revealed no age acceleration, IEAA, or EEAA differences in patients vs controls (Figure S3).

**FIGURE 3:**
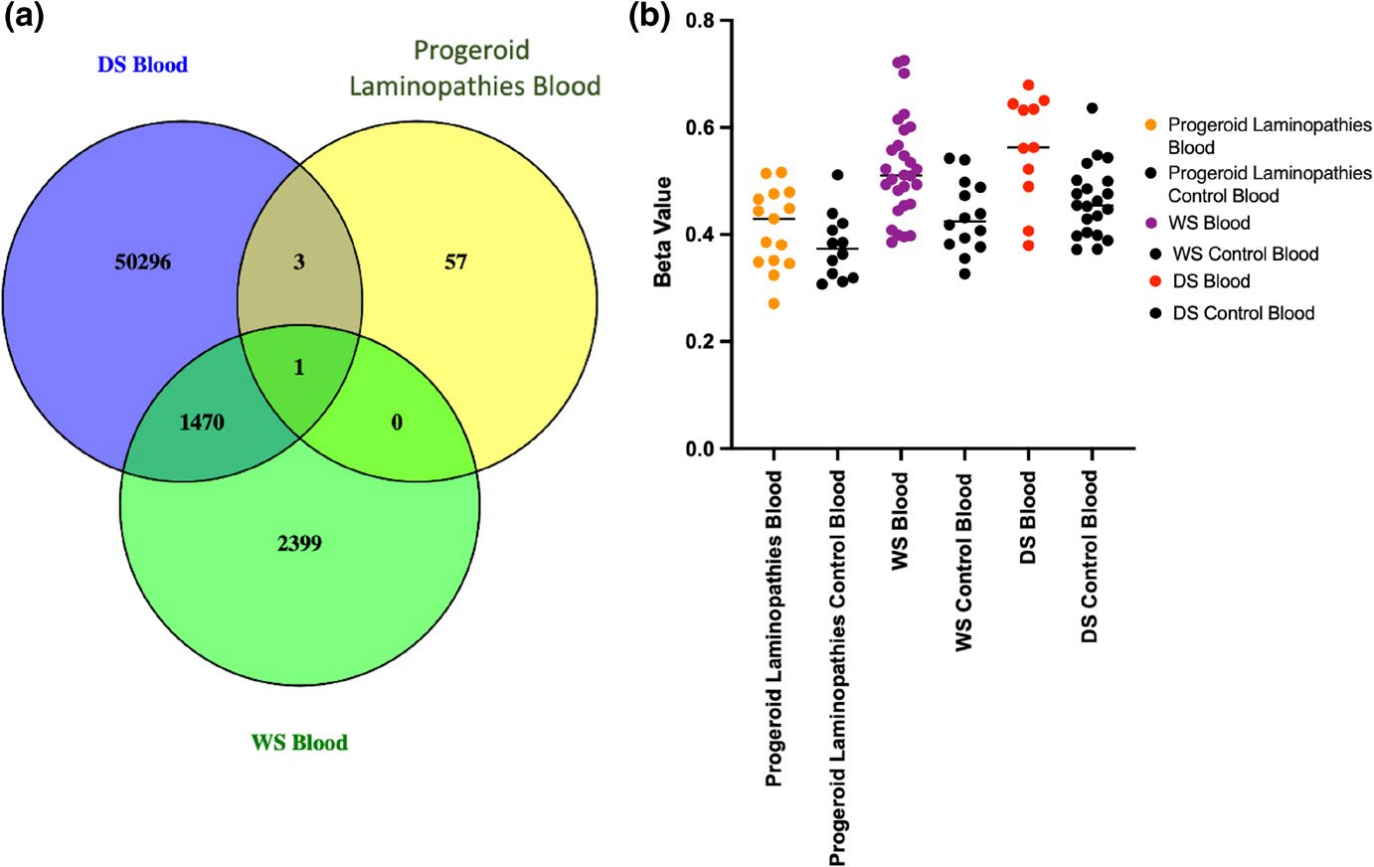
(a) Venn diagram showing overlap of a single genome‐wide significant CpG site (cg06216080) in blood DNA of Hutchinson–Gilford Progeria syndrome (HGPS), Werner syndrome (WS), and Down syndrome (DS) (b) DNA methylation (*β* values) distribution for cg06216080 in all cases and control samples in blood DNA of HGPS, WS, and DS. Median is indicated by a solid black line

We further investigated overlap between the 61 significant CpGs in progeroid laminopathies and differentially methylated CpG sites in the adult progeroid syndrome, Werner syndrome, in the GSE131752 dataset(Maierhofer et al., 2019). This analysis revealed a single common CpG site (cg06216080) with significantly altered DNA methylation in blood DNA of typical WS (Figure 3a). This CpG site is in close vicinity to the transcription start site of the *ENSG00000223989* gene encoding a long non‐coding RNA located on the complementary strand of the 3’ untranslated region of the Catenin Beta Interacting Protein 1 gene (*CTNNBIP1*). We further checked whether cg06216080 is epigenetically altered in patients with Down syndrome (DS) (Haertle et al., 2019), which is considered a segmental progeroid syndrome since DS individuals age prematurely. Similarly, we observed significant hypermethylation at cg06216080 in DS patients when compared to controls (Figure 3a,b). We additionally investigated nearby CpG sites that showed no significant methylation difference following FDR adjustment. Here, we could observe a similar pattern of DNA methylation changes when comparing progeroid laminopathies vs controls across several nearby CpG sites (Figure S4). Therefore, we performed a tiling analysis using a 1 Kb sliding window approach instead of the default 5Kb window in RnBeads. This revealed a 1000 bp region (chr1: 9,908,001–9,909,000) encompassing cg06216080 as well as 3 additional probes with nominal significance following FDR adjustment (*comb.p.adj.fdr* = 0.115, control *β* values mean of means = 0.3713, patient *β* values mean of means = 0.3917). Next, we investigated whether cg06216080 is epigenetically altered during biological aging in an unpublished dataset of 601 healthy controls and 425 individuals with Type 2 diabetes (T2D). Here, cg06216080 methylation displayed no significant correlation with age in healthy controls (*p* value = 0.716) as well as patients with T2D (*p* value = 0.937).

### 2.4 Differential expression analysis of epigenetically altered and interacting genes

Further leveraging publicly available RNA sequencing (RNA‐seq) and expression array datasets, we examined the expression profile of the *ENSG00000223989* long non‐coding RNA (lncRNA) gene in iPSC‐derived smooth muscle cells and primary skin fibroblasts of the HGPS patients as well as patients suffering from *LMNA*‐related dilated cardiomyopathy (DCM) (Table S3). In addition, we measured the expression of *CTNNBIP1* since antisense lncRNAs are known to control the sense gene expression of neighboring protein‐coding genes(Villegas & Zaphiropoulos, 2015). In these cell lines, *ENSG00000223989* lncRNA levels were below the detection limit of our RNA‐seq analysis. Using expression array, we observed a trend of differential *CTNNBIP1* expression between the HGPS SMCs and control SMCs under static conditions (adj. *p* value = 0.06), they did not rise to the statistical significance. Difference of *CTNNBIP1* expression under flow conditions was not significant (adj. *p* value = 0.19). Using RNA‐seq, there was no significant difference of *CTNNBIP1* expressions between HGPS and controls, either in iPSC‐derived smooth muscle cells or in primary fibroblasts (Table S3). These findings suggest that involvement of *ENSG00000223989* lncRNA and *CTNNBIP1* might depend not only on tissue and cell types but also on the conditions (*i*.*e*., static).

## 3 DISCUSSION

In this study, we performed the first genome‐wide DNA methylation analysis in blood DNA of classic HGPS patients and progeroid laminopathy patients harboring the non‐classic *LMNA* mutation. Our analysis revealed DNA methylation aberrations at 61 CpG sites as well as 32 regions after an aggregate analysis on all progeroid laminopathies. These sites were mainly enriched for phosphatidylinositol biosynthetic process, phospholipid biosynthetic process, sarcoplasm, sarcoplasmic reticulum, phosphatase regulator activity, glycerolipid biosynthetic process, glycerophospholipid biosynthetic process, and phosphatidylinositol metabolic process. Interestingly, several of these processes have been associated with normal aging processes previously. Phosphatidylinositol 3‐kinase (PI3K) is part of the PI3K/AKT/mTOR pathway, which has important biological roles in cells (Keppler‐Noreuil et al., 2016; Xie et al., 2019). The PI3K/AKT/mTOR intracellular signaling pathway is involved in cell cycle regulation, thus having key roles in cell proliferation, survival, and metabolism. Furthermore, PI3K as well as its downstream kinases mTOR and S6K has an essential role in aging and longevity in multiple organisms (Bjedov et al., 2010; Harrison et al., 2009; Kenyon, 2005; Morris et al., 1996; Piper et al., 2008; Selman et al., 2009). Phospholipids are the main lipid components of most cellular membranes and are associated with several age‐related diseases (He et al., 2010; Johnson & Stolzing, 2019; Kosicek & Hecimovic, 2013; Liu et al., 2021). Phospholipid metabolism can also potentially regulate age and lifespan. For example, naked mole rats which are known for their long lifespans have a unique membrane phospholipid composition that is hypothesized to be essential to their longevity (Mitchell et al., 2007). Similarly, several of the other enriched pathways have been shown to be associated with aging and age‐related diseases (Jimenez‐Moreno et al., 2008; Liu & Ikegami, 2020; Veeranna et al., 2011), which might indicate a function for those pathways in the observed phenotypes of progeroid laminopathies.

We could not observe DNA methylation differences when comparing probes located in lamin A LADs as well as redistributed lamin A LAD genomic regions. This is in contrast to a recent study by Köhler et al. where DNA methylation alterations were reported in lamin A LADs of primary fibroblasts obtained from classic HGPS patients (Kohler et al., 2020). Köhler et al. mainly focused on LADs specific to the cell type in which they performed differential methylation analysis (fibroblasts). Unfortunately, this was not possible in our analysis due to lack of published lamin A LAD datasets for blood cells. There are variations in LAD organization across various cell types since >70% of LADs are constitutively organized, whereas facultative LADs exhibit cell type‐specific genomic localization (Meuleman et al., 2013; Peric‐Hupkes et al., 2010). Nevertheless, we could observe strong methylation differences in probes located in solo‐WCGW PMDs, which have a high overlap with LADs (Berman et al., 2011). Interestingly, hypomethylation of solo‐WCGW CpG sites in PMDs is associated with chronological age and mitotic cell division (Zhou et al., 2018). Zhou et al. have previously reported (AT)CG(AT) sites, that is., "solo‐WCGW" motifs, in PMDs as a universal indicator of methylation loss due to aging and mitotic cell division in mammalian cells. Therefore, we believe that this difference could be likely due to premature aging or the increased proliferation rate observed in HGPS cells (Bridger & Kill, 2004). This difference was specific to the classic HGPS patients and was not observed in the non‐classic progeroid laminopathy group. CpG sites associated with PMDs were previously reported to be significantly hypermethylated in HGPS fibroblast cells (Kohler et al., 2020), whereas we detected a significant hypomethylation in blood DNA. Therefore, it is important to analyze multiple tissues/cells from patients to better understand disease‐associated epigenetic dysregulation.

One of the highly debated topics is whether aging in HGPS reflects an accelerated form of human aging. Epigenetic clocks are well‐known biomarkers for measuring biological and chronological age in a variety of cells/tissues(Horvath, 2013; Levine et al., 2018; Lu et al., 2019; Salameh et al., 2020). DNA methylation has been also reported to be strongly correlated with aging and mortality across several tissues(Atsem et al., 2016; Fraga et al., 2005; Marioni et al., 2015; Potabattula et al., 2018, 2020; Salameh et al., 2020). Previously, several reports have shown epigenetic age acceleration and DNA methylation alterations to occur in patients with progeroid features including Werner syndrome and Down syndrome (Almenar‐Queralt et al., 2019; El Hajj et al., 2016, 2017; Haertle et al., 2019; Maierhofer et al., 2017). In addition, a recently developed epigenetic clock could observe epigenetic age acceleration in primary fibroblasts of HGPS, whereas the original pan‐tissue epigenetic clock did not identify age acceleration (Horvath et al., 2018). Here, we did not observe epigenetic age acceleration, which might indicate aging processes different to the one measured by the epigenetic clocks. Nevertheless, we could observe a common epigenetically dysregulated region in progeroid laminopathies as well as the segmental progeroid syndromes, Werner syndrome (also known as adult progeria), and Down syndrome. This region is in near vicinity to a transcription start site of a lncRNA positioned anti‐sense to the Catenin Beta Interacting Protein 1 gene (*CTNNBIP1*), an antagonist of Wnt signaling. Anti‐sense lncRNA is transcribed from the opposite DNA strand to that of the sense transcript of genes and can function in cis or in trans (Pelechano & Steinmetz, 2013). In addition, anti‐sense transcripts can regulate the transcription of sense transcripts via transcriptional interference (Faghihi & Wahlestedt, 2009). *CTNNBIP1* encodes beta‐catenin interacting protein 1 (ICAT), which prevents the interaction between TCF4 and β‐catenin (Tago et al., 2000). Interestingly, Wnt signaling is reported by Hernandez et al. to be decreased in both progeric mouse and human cells (Hernandez et al., 2010). Similar observations of reduced Wnt signaling were also observed in Down syndrome patients(Granno et al., 2019). Expression analysis revealed no significant *CTNNBIP1* transcriptional changes in several of the analyzed HGPS tissues. This may be in part explained by the finding that expression of the lncRNA *ENSG00000223989* was below the threshold in those tissues including smooth muscle cells, cardiac myocytes, and fibroblast. Our observation that methylation levels at cg06216080 are not associated with chronological age in healthy controls and diabetic individuals indicates that differential methylation at this CpG site is not related to normal aging processes. However, additional experiments are needed to determine the function of this lncRNA and in which stage of development or tissue it is transcribed.

## 4 CONCLUSION

To date, most studies on epigenetic alterations in HGPS have focused on primary fibroblast cells. This is the first study to measure DNA methylation alterations in blood DNA of classic HGPS patients and non‐classical progeroid laminopathies. Interestingly, we observed significant hypomethylation at solo‐WCGW CpG sites in PMDs for HGPS patients; however, we detected no epigenetic age acceleration. Collectively, our results indicate minor methylation differences in progeroid laminopathy patients when compared with controls as well as accelerated aging independent of the biological aging processes measured by epigenetic clocks.

## 5 MATERIALS AND METHODS

### 5.1 Study samples

Whole blood DNA samples of 15 patients with progeroid laminopathies were obtained from the Progeria Research foundation (PRF) blood and tissue bank (Table S4). In total, DNA methylation was measured in eight classical HGPS (HGABLDNA146, HGABLDNA306, HGABLDNA331, HGABLDNA352, HGABLDNA378, UHGABLDNA172, UHGABLDNA351, UHGABLDNA480) and seven progeroid laminopathy patients with the non‐classical *LMNA* mutation (PSABLDNA199, PSABLDNA295, PSABLDNA316, PSABLDNA379, PSABLDNA406, PSABLDNA501, PSABLDNA531) as well as 12 control samples. The study was approved by the Institutional Review Board (IRB) of Qatar Biomedical Research Institute (2019–029) as well as the Progeria research foundation the Rhode Island Hospital Committee on the Protection of Human Subjects, Federal Wide Assurance FWA00001230, study CMTT#0146‐09 and the University of Michigan FWA00004969, IRB00001996.

### 5.2 DNA methylation quantification

Genomic DNA was bisulfite converted via the EZ DNA Methylation™ Kit (Zymo Research, Irvine, CA, USA). Following whole‐genome amplification and enzymatic fragmentation, DNA methylation levels were quantified using the Infinium MethylationEPIC BeadChips kit (Illumina) according to the manufacturer's instructions. All samples were processed simultaneously to avoid batch effects. Furthermore, cases and controls were randomly hybridized on the array to reduce positional effect bias. BeadChips were scanned using an Illumina iScan, and raw intensity data files (IDAT) were exported to R software package. Analysis of the IDAT files was performed in R using the RnBeads package (Muller et al., 2019). Quality control and preprocessing steps involved the following: (i) Filtering out probes overlapping SNPs (*n* = 17,371), (ii) Filtering out probes and/or samples with the highest fraction of unreliable measurements using greedycut (*n* = 2379); in total, 19750 probes were removed and all samples were retained, and (iii) Subsequently, data normalization was performed using Dasen, followed by an additional filtering step to remove probes located on sex chromosomes (*n* = 18,986). Overall, 825,177 probes were retained for further differential DNA methylation analysis. The relative proportion of white blood counts was estimated using the Houseman et al. method (2014). This method is based on blood‐derived DNA methylation signatures measured using the Illumina HumanMethylationEPIC array, which can be used to estimate the proportions of neutrophil, monocyte, B‐lymphocyte, natural killer, and CD4+ and CD8+ T‐cell fractions.

### 5.3 Differential DNA methylation analysis in progeroid laminopathies

Differential methylation analysis was conducted at the CpG site and region level. Cellular heterogeneity was accounted for in the profiled samples using the RefFreeEWAS method^5^ followed by limma‐based analysis to adjust for covariates. At the region level, differential methylation was quantified using several metrics including analyzing the following quantities for each region: the mean difference in means across all sites in a region of the two groups being compared and the mean of quotients in mean methylation as well as a combined p value was calculated from all site p values in the region. The p values were corrected for multiple testing via the false discovery rate (FDR) method. Genomic regions were defined as follows: tiling (5 kb), genes, promoters, and CpG islands. Previously reported coordinates of “solo‐WCGWs” CpGs (Zhou et al., 2018) and lamin A and B LADs (Guelen et al., 2008; Lund et al., 2015) were used to test for methylation level differences across those regions between HGPS and control samples and significance of methylation differences calculated using Welch's two‐sample t test. To check possible regulatory mechanisms underlying the significant associated CpG sites, a quantitative trait methylation test was conducted using the BIOS QTL browser.

### 5.4 Differential DNA methylation analysis of cg06216080

A total of 1026 blood DNA samples, out of which 425 are T2D patients, were collected from Qatar BioBank (QBB) and profiled for DNA methylation using Illumina EPIC arrays in 3 batches. A linear regression model was used to compute the association between cg06216080 and age in each of controls and T2D cases, separately. Controls and cases were divided each into 3 groups based on BMI (lean/normal, overweight, and obese) that were tested separately for association with cg06216080. Controls and cases were also tested for association with cg06216080 above specific age ranges (50, 55, 60, and 65). Covariates that were added to the model are BMI, gender, batch effect, well position, plate number, measured cell counts (neutrophils, basophils, eosinophils, lymphocytes, monocytes), smoking surrogate (AHRR), and four principal components from genomic data to correct for population stratification.

### 5.5 Expression analysis of publicly available datasets

For expression analysis, several publicly available array and RNA‐seq datasets were used to investigate the association of DNA methylation alterations with gene expression changes. Table S5 shows the dataset and samples analyzed. GEO2R was used to analyze array profiled data using GEOquery and limmaR packages from the Bioconductor project. Results generated by GEO2R are presented as a table of genes ordered by significance, and as a collection of graphic plots to help visualize differentially expressed genes and assess data set quality. CLC Genomics Workbench was used to analyze RNA‐seq and to detect the lncRNA gene expression. Fastq file quality was checked using FastQC and afterward aligned to the hg19 human reference genome in CLC Genomics Workbench (Qiagen) using default settings. The abundance of transcripts was measured as the score of TPM (transcripts per million) and subsequently subjected to differential gene expression.

## CONFLICT OF INTEREST

None declared.

## AUTHOR CONTRIBUTIONS

Y.B., A.R., R.P., and A.Q. performed experiments. Y.B., A.R., T.A., and S.H. performed bioinformatic analyses. N.A.Y performed the Qatar BioBank data analysis. J.O., G.M., and T.H. collected study samples. Y.B., A.M., H.F.H., S.H., and N.E.H wrote the manuscript. A.M., H.F.H., S.H., and N.E.H. critically reviewed and edited the manuscript. N.E.H designed the study.

## Supporting information

Table S1

Table S2

Fig S1

Fig S2

Fig S3

Fig S4

## Data Availability

The IDAT files generated during this study are deposited in the Gene Expression Omnibus (GEO accession: GSE182991).

## References

[acel13555-bib-0001] Almenar‐Queralt, A. , Merkurjev, D. , Kim, H. S. , Navarro, M. , Ma, Q. I. , Chaves, R. S. , Allegue, C. , Driscoll, S. P. , Chen, A. G. , Kohlnhofer, B. , Fong, L. K. , Woodruff, G. , Mackintosh, C. , Bohaciakova, D. , Hruska‐Plochan, M. , Tadokoro, T. , Young, J. E. , El Hajj, N. , Dittrich, M. , … Garcia‐Bassets, I. (2019). Chromatin establishes an immature version of neuronal protocadherin selection during the naive‐to‐primed conversion of pluripotent stem cells. Nature Genetics, 51(12), 1691–1701. 10.1038/s41588-019-0526-4 31740836 PMC7061033

[acel13555-bib-0002] Atsem, S. , Reichenbach, J. , Potabattula, R. , Dittrich, M. , Nava, C. , Depienne, C. , Böhm, L. , Rost, S. , Hahn, T. , Schorsch, M. , & Haaf, T. & El Hajj, N. (2016). Paternal age effects on sperm FOXK1 and KCNA7 methylation and transmission into the next generation. Human Molecular Genetics, 25(22), 4996–5005. 10.1093/hmg/ddw328 28171595 PMC5418740

[acel13555-bib-0003] Berman, B. P. , Weisenberger, D. J. , Aman, J. F. , Hinoue, T. , Ramjan, Z. , Liu, Y. , Noushmehr, H. , Lange, C. P. E. , van Dijk, C. M. , Tollenaar, R. A. E. M. , Van Den Berg, D. , & Laird, P. W. (2011). Regions of focal DNA hypermethylation and long‐range hypomethylation in colorectal cancer coincide with nuclear lamina‐associated domains. Nature Genetics, 44(1), 40–46. 10.1038/ng.969 22120008 PMC4309644

[acel13555-bib-0004] Bjedov, I. , Toivonen, J. M. , Kerr, F. , Slack, C. , Jacobson, J. , Foley, A. , & Partridge, L. (2010). Mechanisms of life span extension by rapamycin in the fruit fly *Drosophila melanogaster* . Cell Metabolism, 11(1), 35–46. 10.1016/j.cmet.2009.11.010 20074526 PMC2824086

[acel13555-bib-0005] Breeze, C. E. , Reynolds, A. P. , van Dongen, J. , Dunham, I. , Lazar, J. , Neph, S. , Vierstra, J. , Bourque, G. , Teschendorff, A. E. , Stamatoyannopoulos, J. A. , & Beck, S. (2019). eFORGE v2.0: updated analysis of cell type‐specific signal in epigenomic data. Bioinformatics, 35(22), 4767–4769. 10.1093/bioinformatics/btz456 31161210 PMC6853678

[acel13555-bib-0006] Bridger, J. M. , & Kill, I. R. (2004). Aging of Hutchinson‐Gilford progeria syndrome fibroblasts is characterised by hyperproliferation and increased apoptosis. Experimental Gerontology, 39(5), 717–724. 10.1016/j.exger.2004.02.002 15130666

[acel13555-bib-0007] Burke, B. , & Stewart, C. L. (2006). The laminopathies: The functional architecture of the nucleus and its contribution to disease. Annual Review of Genomics and Human Genetics, 7, 369–405. 10.1146/annurev.genom.7.080505.115732 16824021

[acel13555-bib-0008] Cheedipudi, S. M. , Matkovich, S. J. , Coarfa, C. , Hu, X. , Robertson, M. J. , Sweet, M. , Taylor, M. , Mestroni, L. , Cleveland, J. , Willerson, J. T. , Gurha, P. , & Marian, A. J. (2019). Genomic reorganization of lamin‐associated domains in cardiac myocytes is associated with differential gene expression and DNA methylation in human dilated cardiomyopathy. Circulation Research, 124(8), 1198–1213. 10.1161/CIRCRESAHA.118.314177 30739589 PMC6459729

[acel13555-bib-0009] Davies, B. S. , Coffinier, C. , Yang, S. H. , Barnes, R. H. 2nd , Jung, H. J. , Young, S. G. , & Fong, L. G. (2011). Investigating the purpose of prelamin A processing. Nucleus, 2(1), 4–9. 10.1093/hmg/ddq158 21647293 PMC3104803

[acel13555-bib-0010] de Leeuw, R. , Gruenbaum, Y. , & Medalia, O. (2018). Nuclear lamins: thin filaments with major functions. Trends in Cell Biology, 28(1), 34–45. 10.1016/j.tcb.2017.08.004 28893461

[acel13555-bib-0011] De Sandre‐Giovannoli, A. , Bernard, Rafaëlle , Cau, P. , Navarro, C. , Amiel, J. , Boccaccio, Irène , Lyonnet, S. , Stewart, C. L. , Munnich, A. , Le Merrer, M. , & Lévy, N. (2003). Lamin a truncation in Hutchinson‐Gilford progeria. Science, 300(5628), 2055. 10.1126/science.1084125 12702809

[acel13555-bib-0012] Dixon, J. R. , Selvaraj, S. , Yue, F. , Kim, A. , Li, Y. , Shen, Y. , Hu, M. , Liu, J. S. , & Ren, B. (2012). Topological domains in mammalian genomes identified by analysis of chromatin interactions. Nature, 485(7398), 376–380. 10.1038/nature11082 22495300 PMC3356448

[acel13555-bib-0013] El Hajj, N. , Dittrich, M. , Böck, J. , Kraus, T. F. J. , Nanda, I. , Müller, T. , Seidmann, L. , Tralau, T. , Galetzka, D. , Schneider, E. , & Haaf, T. (2016). Epigenetic dysregulation in the developing Down syndrome cortex. Epigenetics, 11(8), 563–578. 10.1080/15592294.2016.1192736 27245352 PMC4990229

[acel13555-bib-0014] El Hajj, N. , Dittrich, M. , & Haaf, T. (2017). Epigenetic dysregulation of protocadherins in human disease. Seminars in Cell & Developmental Biology, 69, 172–182. 10.1016/j.semcdb.2017.07.007 28694114

[acel13555-bib-0015] Eriksson, M. , Brown, W. T. , Gordon, L. B. , Glynn, M. W. , Singer, J. , Scott, L. , Erdos, M. R. , Robbins, C. M. , Moses, T. Y. , Berglund, P. , Dutra, A. , Pak, E. , Durkin, S. , Csoka, A. B. , Boehnke, M. , Glover, T. W. , & Collins, F. S. (2003). Recurrent de novo point mutations in lamin A cause Hutchinson‐Gilford progeria syndrome. Nature, 423(6937), 293–298. 10.1038/nature01629 12714972 PMC10540076

[acel13555-bib-0016] Faghihi, M. A. , & Wahlestedt, C. (2009). Regulatory roles of natural antisense transcripts. Nature Reviews Molecular Cell Biology, 10(9), 637–643. 10.1038/nrm2738 19638999 PMC2850559

[acel13555-bib-0017] Fraga, M. F. , Ballestar, E. , Paz, M. F. , Ropero, S. , Setien, F. , Ballestar, M. L. , & Esteller, M. (2005). Epigenetic differences arise during the lifetime of monozygotic twins. Proceedings of the National Academy of Sciences of the United States of America, 102(30), 10604–10609. 10.1073/pnas.0500398102 16009939 PMC1174919

[acel13555-bib-0018] Granno, S. , Nixon‐Abell, J. , Berwick, D. C. , Tosh, J. , Heaton, G. , Almudimeegh, S. , Nagda, Z. , Rain, J.‐C. , Zanda, M. , Plagnol, V. , Tybulewicz, V. L. J. , Cleverley, K. , Wiseman, F. K. , Fisher, E. M. C. , & Harvey, K. (2019). Downregulated Wnt/beta‐catenin signalling in the Down syndrome hippocampus. Scientific Reports, 9(1), 7322. 10.1038/s41598-019-43820-4 31086297 PMC6513850

[acel13555-bib-0019] Guelen, L. , Pagie, L. , Brasset, E. , Meuleman, W. , Faza, M. B. , Talhout, W. , Eussen, B. H. , de Klein, A. , Wessels, L. , de Laat, W. , & van Steensel, B. (2008). Domain organization of human chromosomes revealed by mapping of nuclear lamina interactions. Nature, 453(7197), 948–951. 10.1038/nature06947 18463634

[acel13555-bib-0020] Haertle, L. , Müller, T. , Lardenoije, R. , Maierhofer, A. , Dittrich, M. , Riemens, R. J. M. , Stora, S. , Roche, M. , Leber, M. , Riedel‐Heller, S. , Wagner, M. , Scherer, M. , Ravel, A. , Mircher, C. , Cieuta‐Walti, C. , Durand, S. , van de Hove, D. L. A. , Hoffmann, P. , Ramirez, A. , … Mégarbané, A. (2019). Methylomic profiling in trisomy 21 identifies cognition‐ and Alzheimer's disease‐related dysregulation. Clinical Epigenetics, 11(1), 195. 10.1186/s13148-019-0787-x 31843015 PMC6916110

[acel13555-bib-0021] Hansen, K. D. , Sabunciyan, S. , Langmead, B. , Nagy, N. , Curley, R. , Klein, G. , Klein, E. , Salamon, D. , & Feinberg, A. P. (2014). Large‐scale hypomethylated blocks associated with Epstein‐Barr virus‐induced B‐cell immortalization. Genome Research, 24(2), 177–184. 10.1101/gr.157743.113 24068705 PMC3912409

[acel13555-bib-0022] Harrison, D. E. , Strong, R. , Sharp, Z. D. , Nelson, J. F. , Astle, C. M. , Flurkey, K. , Nadon, N. L. , Wilkinson, J. E. , Frenkel, K. , Carter, C. S. , Pahor, M. , Javors, M. A. , Fernandez, E. , & Miller, R. A. (2009). Rapamycin fed late in life extends lifespan in genetically heterogeneous mice. Nature, 460(7253), 392–395. 10.1038/nature08221 19587680 PMC2786175

[acel13555-bib-0023] He, X. , Huang, Y. , Li, B. , Gong, C. X. , & Schuchman, E. H. (2010). Deregulation of sphingolipid metabolism in Alzheimer's disease. Neurobiology of Aging, 31(3), 398–408. 10.1016/j.neurobiolaging.2008.05.010 18547682 PMC2829762

[acel13555-bib-0024] Hennekam, R. C. (2006). Hutchinson‐Gilford progeria syndrome: review of the phenotype. American Journal of Medical Genetics. Part A, 140(23), 2603–2624. 10.1002/ajmg.a.31346 16838330

[acel13555-bib-0025] Hernandez, L. , Roux, K. J. , Wong, E. S. M. , Mounkes, L. C. , Mutalif, R. , Navasankari, R. , Rai, B. , Cool, S. , Jeong, J.‐W. , Wang, H. , Lee, H.‐S. , Kozlov, S. , Grunert, M. , Keeble, T. , Jones, C. M. , Meta, M. D. , Young, S. G. , Daar, I. O. , Burke, B. , … Stewart, C. L. (2010). Functional coupling between the extracellular matrix and nuclear lamina by Wnt signaling in progeria. Developmental Cell, 19(3), 413–425. 10.1016/j.devcel.2010.08.013 20833363 PMC2953243

[acel13555-bib-0026] Heyn, H. , Moran, S. , & Esteller, M. (2013). Aberrant DNA methylation profiles in the premature aging disorders Hutchinson‐Gilford Progeria and Werner syndrome. Epigenetics, 8(1), 28–33. 10.4161/epi.23366 23257959 PMC3549877

[acel13555-bib-0027] Horvath, S. (2013). DNA methylation age of human tissues and cell types. Genome Biology, 14(10), R115. 10.1186/gb-2013-14-10-r115 24138928 PMC4015143

[acel13555-bib-0028] Horvath, S. , Oshima, J. , Martin, G. M. , Lu, A. T. , Quach, A. , Cohen, H. , Felton, S. , Matsuyama, M. , Lowe, D. , Kabacik, S. , Wilson, J. G. , Reiner, A. P. , Maierhofer, A. , Flunkert, J. , Aviv, A. , Hou, L. , Baccarelli, A. A. , Li, Y. , Stewart, J. D. , … Raj, K. (2018). Epigenetic clock for skin and blood cells applied to Hutchinson Gilford Progeria Syndrome and ex vivo studies. Aging (Albany NY), 10(7), 1758–1775. 10.18632/aging.101508 30048243 PMC6075434

[acel13555-bib-0029] Houseman, E. A. , Molitor, J. , & Marsit, C. J. (2014). Reference‐free cell mixture adjustments in analysis of DNA methylation data. Bioinformatics, 30(10), 1431–1439. 10.1093/bioinformatics/btu029 24451622 PMC4016702

[acel13555-bib-0030] Jimenez‐Moreno, R. , Wang, Z. M. , Gerring, R. C. , & Delbono, O. (2008). Sarcoplasmic reticulum Ca2+ release declines in muscle fibers from aging mice. Biophysical Journal, 94(8), 3178–3188. 10.1529/biophysj.107.118786 18178643 PMC2275691

[acel13555-bib-0031] Johnson, A. A. , & Stolzing, A. (2019). The role of lipid metabolism in aging, lifespan regulation, and age‐related disease. Aging Cell, 18(6), e13048. 10.1111/acel.13048 31560163 PMC6826135

[acel13555-bib-0032] Kenyon, C. (2005). The plasticity of aging: insights from long‐lived mutants. Cell, 120(4), 449–460. 10.1016/j.cell.2005.02.002 15734678

[acel13555-bib-0033] Keppler‐Noreuil, K. M. , Parker, V. E. , Darling, T. N. , & Martinez‐Agosto, J. A. (2016). Somatic overgrowth disorders of the PI3K/AKT/mTOR pathway & therapeutic strategies. American Journal of Medical Genetics Part C Seminars in Medical Genetics, 172(4), 402–421. 10.1002/ajmg.c.31531 PMC5592089 27860216

[acel13555-bib-0034] Köhler, F. , Bormann, F. , Raddatz, G. , Gutekunst, J. , Corless, S. , Musch, T. , Lonsdorf, A. S. , Erhardt, S. , Lyko, F. , & Rodríguez‐Paredes, M. (2020). Epigenetic deregulation of lamina‐associated domains in Hutchinson‐Gilford progeria syndrome. Genome Medicine, 12(1), 46. 10.1186/s13073-020-00749-y 32450911 PMC7249329

[acel13555-bib-0035] Kosicek, M. , & Hecimovic, S. (2013). Phospholipids and Alzheimer's disease: Alterations, mechanisms and potential biomarkers. International Journal of Molecular Sciences, 14(1), 1310–1322. 10.3390/ijms14011310 23306153 PMC3565322

[acel13555-bib-0036] Levine, M. E. , Lu, A. T. , Quach, A. , Chen, B. H. , Assimes, T. L. , Bandinelli, S. , Hou, L. , Baccarelli, A. A. , Stewart, J. D. , Li, Y. , Whitsel, E. A. , Wilson, J. G. , Reiner, A. P. , Aviv, A. , Lohman, K. , Liu, Y. , Ferrucci, L. , & Horvath, S. (2018). An epigenetic biomarker of aging for lifespan and healthspan. Aging (Albany NY), 10(4), 573–591. 10.18632/aging.101414 29676998 PMC5940111

[acel13555-bib-0037] Lieberman‐Aiden, E. , van Berkum, N. L. , Williams, L. , Imakaev, M. , Ragoczy, T. , Telling, A. , Amit, I. , Lajoie, B. R. , Sabo, P. J. , Dorschner, M. O. , Sandstrom, R. , Bernstein, B. , Bender, M. A. , Groudine, M. , Gnirke, A. , Stamatoyannopoulos, J. , Mirny, L. A. , Lander, E. S. , & Dekker, J. (2009). Comprehensive mapping of long‐range interactions reveals folding principles of the human genome. Science, 326(5950), 289–293. 10.1126/science.1181369 19815776 PMC2858594

[acel13555-bib-0038] Liu, G.‐H. , Barkho, B. Z. , Ruiz, S. , Diep, D. , Qu, J. , Yang, S.‐L. , Panopoulos, A. D. , Suzuki, K. , Kurian, L. , Walsh, C. , Thompson, J. , Boue, S. , Fung, H. L. , Sancho‐Martinez, I. , Zhang, K. , Iii, J. Y. , & Belmonte, J. C. I. (2011). Recapitulation of premature ageing with iPSCs from Hutchinson‐Gilford progeria syndrome. Nature, 472(7342), 221–225. 10.1038/nature09879 21346760 PMC3088088

[acel13555-bib-0039] Liu, S. Y. , & Ikegami, K. (2020). Nuclear lamin phosphorylation: An emerging role in gene regulation and pathogenesis of laminopathies. Nucleus, 11(1), 299–314. 10.1080/19491034.2020.1832734 33030403 PMC7588210

[acel13555-bib-0040] Liu, T. T. , Pang, S. J. , Jia, S. S. , Man, Q. Q. , Li, Y. Q. , Song, S. , & Zhang, J. (2021). Association of plasma phospholipids with age‐related cognitive impairment: Results from a cross‐sectional study. Nutrients, 13(7), 2185. 10.3390/nu13072185 34201969 PMC8308406

[acel13555-bib-0041] Lochs, S. J. A. , Kefalopoulou, S. , & Kind, J. (2019). Lamina associated domains and gene regulation in development and cancer. Cells, 8(3), 271. 10.3390/cells8030271 PMC6468596 30901978

[acel13555-bib-0042] Lu, A. T. , Quach, A. , Wilson, J. G. , Reiner, A. P. , Aviv, A. , Raj, K. , Hou, L. , Baccarelli, A. A. , Li, Y. , Stewart, J. D. , Whitsel, E. A. , Assimes, T. L. , Ferrucci, L. , & Horvath, S. (2019). DNA methylation GrimAge strongly predicts lifespan and healthspan. Aging (Albany NY), 11(2), 303–327. 10.18632/aging.101684 30669119 PMC6366976

[acel13555-bib-0043] Lund, E. G. , Duband‐Goulet, I. , Oldenburg, A. , Buendia, B. , & Collas, P. (2015). Distinct features of lamin A‐interacting chromatin domains mapped by ChIP‐sequencing from sonicated or micrococcal nuclease‐digested chromatin. Nucleus, 6(1), 30–39. 10.4161/19491034.2014.990855 25602132 PMC4615303

[acel13555-bib-0044] Lund, E. , Oldenburg, A. R. , & Collas, P. (2014). Enriched domain detector: a program for detection of wide genomic enrichment domains robust against local variations. Nucleic Acids Research, 42(11), e92. 10.1093/nar/gku324 24782521 PMC4066758

[acel13555-bib-0045] Maierhofer, A. , Flunkert, J. , Oshima, J. , Martin, G. M. , Haaf, T. , & Horvath, S. (2017). Accelerated epigenetic aging in Werner syndrome. Aging (Albany NY), 9(4), 1143–1152. 10.18632/aging.101217 28377537 PMC5425119

[acel13555-bib-0046] Maierhofer, A. , Flunkert, J. , Oshima, J. , Martin, G. M. , Poot, M. , Nanda, I. , Dittrich, M. , Müller, T. , & Haaf, T. (2019). Epigenetic signatures of Werner syndrome occur early in life and are distinct from normal epigenetic aging processes. Aging Cell, 18(5), e12995. 10.1111/acel.12995 31259468 PMC6718529

[acel13555-bib-0047] Marioni, R. E. , Shah, S. , McRae, A. F. , Chen, B. H. , Colicino, E. , Harris, S. E. , Gibson, J. , Henders, A. K. , Redmond, P. , Cox, S. R. , Pattie, A. , Corley, J. , Murphy, L. , Martin, N. G. , Montgomery, G. W. , Feinberg, A. P. , Fallin, M. D. , Multhaup, M. L. , Jaffe, A. E. , … Deary, I. J. (2015). DNA methylation age of blood predicts all‐cause mortality in later life. Genome Biology, 16, 25. 10.1186/s13059-015-0584-6 25633388 PMC4350614

[acel13555-bib-0048] McCord, R. P. , Nazario‐Toole, A. , Zhang, H. , Chines, P. S. , Zhan, Y. E. , Erdos, M. R. , Collins, F. S. , Dekker, J. , & Cao, K. (2013). Correlated alterations in genome organization, histone methylation, and DNA‐lamin A/C interactions in Hutchinson‐Gilford progeria syndrome. Genome Research, 23(2), 260–269. 10.1101/gr.138032.112 23152449 PMC3561867

[acel13555-bib-0049] Meuleman, W. , Peric‐Hupkes, D. , Kind, J. , Beaudry, J.‐B. , Pagie, L. , Kellis, M. , Reinders, M. , Wessels, L. , & van Steensel, B. (2013). Constitutive nuclear lamina‐genome interactions are highly conserved and associated with A/T‐rich sequence. Genome Research, 23(2), 270–280. 10.1101/gr.141028.112 23124521 PMC3561868

[acel13555-bib-0050] Mitchell, T. W. , Buffenstein, R. , & Hulbert, A. J. (2007). Membrane phospholipid composition may contribute to exceptional longevity of the naked mole‐rat (*Heterocephalus glaber*): A comparative study using shotgun lipidomics. Experimental Gerontology, 42(11), 1053–1062. 10.1016/j.exger.2007.09.004 18029129

[acel13555-bib-0051] Morris, J. Z. , Tissenbaum, H. A. , & Ruvkun, G. (1996). A phosphatidylinositol‐3‐OH kinase family member regulating longevity and diapause in *Caenorhabditis elegans* . Nature, 382(6591), 536–539. 10.1038/382536a0 8700226

[acel13555-bib-0052] Muller, F. , Scherer, M. , Assenov, Y. , Lutsik, P. , Walter, J. , Lengauer, T. , & Bock, C. (2019). RnBeads 2.0: comprehensive analysis of DNA methylation data. Genome Biology, 20(1), 55. 10.1186/s13059-019-1664-9 30871603 PMC6419383

[acel13555-bib-0053] Pelechano, V. , & Steinmetz, L. M. (2013). Gene regulation by antisense transcription. Nature Reviews Genetics, 14(12), 880–893. 10.1038/nrg3594 24217315

[acel13555-bib-0054] Peric‐Hupkes, D. , Meuleman, W. , Pagie, L. , Bruggeman, S. W. M. , Solovei, I. , Brugman, W. , Gräf, S. , Flicek, P. , Kerkhoven, R. M. , van Lohuizen, M. , Reinders, M. , Wessels, L. , & van Steensel, B. (2010). Molecular maps of the reorganization of genome‐nuclear lamina interactions during differentiation. Molecular Cell, 38(4), 603–613. 10.1016/j.molcel.2010.03.016 20513434 PMC5975946

[acel13555-bib-0055] Piper, M. D. , Selman, C. , McElwee, J. J. , & Partridge, L. (2008). Separating cause from effect: how does insulin/IGF signalling control lifespan in worms, flies and mice? Journal of Internal Medicine, 263(2), 179–191. 10.1111/j.1365-2796.2007.01906.x 18226095

[acel13555-bib-0056] Potabattula, R. , Dittrich, M. , Böck, J. , Haertle, L. , Müller, T. , Hahn, T. , Schorsch, M. , Hajj, N. E. , & Haaf, T. (2018). Allele‐specific methylation of imprinted genes in fetal cord blood is influenced by cis‐acting genetic variants and parental factors. Epigenomics, 10(10), 1315–1326. 10.2217/epi-2018-0059 30238782 PMC6240887

[acel13555-bib-0057] Potabattula, R. , Zacchini, F. , Ptak, G. E. , Dittrich, M. , Müller, T. , El Hajj, N. , Hahn, T. , Drummer, C. , Behr, R. , Lucas‐Hahn, A. , Niemann, H. , Schorsch, M. , & Haaf, T. (2020). Increasing methylation of sperm rDNA and other repetitive elements in the aging male mammalian germline. Aging Cell, 19(8), e13181. 10.1111/acel.13181 32608562 PMC7431825

[acel13555-bib-0058] Ren, X. , & Kuan, P. F. (2019). methylGSA: A Bioconductor package and Shiny app for DNA methylation data length bias adjustment in gene set testing. Bioinformatics, 35(11), 1958–1959. 10.1093/bioinformatics/bty892 30346483

[acel13555-bib-0059] Salameh, Y. , Bejaoui, Y. , & El Hajj, N. (2020). DNA methylation biomarkers in aging and age‐related diseases. Frontiers in Genetics, 11, 171. 10.3389/fgene.2020.00171 32211026 PMC7076122

[acel13555-bib-0060] Selman, C. , Tullet, J. M. A. , Wieser, D. , Irvine, E. , Lingard, S. J. , Choudhury, A. I. , Claret, M. , Al‐Qassab, H. , Carmignac, D. , Ramadani, F. , Woods, A. , Robinson, I. C. A. , Schuster, E. , Batterham, R. L. , Kozma, S. C. , Thomas, G. , Carling, D. , Okkenhaug, K. , Thornton, J. M. , … Withers, D. J. (2009). Ribosomal protein S6 kinase 1 signaling regulates mammalian life span. Science, 326(5949), 140–144. 10.1126/science.1177221 19797661 PMC4954603

[acel13555-bib-0061] Shumaker, D. K. , Dechat, T. , Kohlmaier, A. , Adam, S. A. , Bozovsky, M. R. , Erdos, M. R. , Eriksson, M. , Goldman, A. E. , Khuon, S. , Collins, F. S. , Jenuwein, T. , & Goldman, R. D. (2006). Mutant nuclear lamin A leads to progressive alterations of epigenetic control in premature aging. Proceedings of the National Academy of Sciences of the United States of America, 103(23), 8703–8708. 10.1073/pnas.0602569103 16738054 PMC1472659

[acel13555-bib-0062] Sinensky, M. , Fantle, K. , Trujillo, M. , McLain, T. , Kupfer, A. , & Dalton, M. (1994). The processing pathway of prelamin A. Journal of Cell Science, 107(Pt 1), 61–67. 10.1242/jcs.107.1.61 8175923

[acel13555-bib-0063] Tago, K. , Nakamura, T. , Nishita, M. , Hyodo, J. , Nagai, S. , Murata, Y. , & Akiyama, T. (2000). Inhibition of Wnt signaling by ICAT, a novel beta‐catenin‐interacting protein. Genes & Development, 14(14), 1741–1749. 10898789 PMC316784

[acel13555-bib-0064] Veeranna , Yang, D.‐S. , Lee, J.‐H. , Vinod, K. Y. , Stavrides, P. , Amin, N. D. , Pant, H. C. , & Nixon, R. A. (2011). Declining phosphatases underlie aging‐related hyperphosphorylation of neurofilaments. Neurobiology of Aging, 32(11), 2016–2029. 10.1016/j.neurobiolaging.2009.12.001 20031277 PMC2891331

[acel13555-bib-0065] Vidak, S. , & Foisner, R. (2016). Molecular insights into the premature aging disease progeria. Histochemistry and Cell Biology, 145(4), 401–417. 10.1007/s00418-016-1411-1 26847180 PMC4796323

[acel13555-bib-0066] Villegas, V. E. , & Zaphiropoulos, P. G. (2015). Neighboring gene regulation by antisense long non‐coding RNAs. International Journal of Molecular Sciences, 16(2), 3251–3266. 10.3390/ijms16023251 25654223 PMC4346893

[acel13555-bib-0067] Wong, X. , & Stewart, C. L. (2020). The Laminopathies and the insights they provide into the structural and functional organization of the nucleus. Annual Review of Genomics and Human Genetics, 21, 263–288. 10.1146/annurev-genom-121219-083616 32428417

[acel13555-bib-0068] Worman, H. J. (2012). Nuclear lamins and laminopathies. The Journal of Pathology, 226(2), 316–325. 10.1002/path.2999 21953297 PMC6673656

[acel13555-bib-0069] Xie, Y. , Shi, X. , Sheng, K. , Han, G. , Li, W. , Zhao, Q. , Jiang, B. , Feng, J. , Li, J. , & Gu, Y. (2019). PI3K/Akt signaling transduction pathway, erythropoiesis and glycolysis in hypoxia (Review). Molecular Medicine Reports, 19(2), 783–791. 10.3892/mmr.2018.9713 30535469 PMC6323245

[acel13555-bib-0070] Zhou, W. , Dinh, H. Q. , Ramjan, Z. , Weisenberger, D. J. , Nicolet, C. M. , Shen, H. , Laird, P. W. , & Berman, B. P. (2018). DNA methylation loss in late‐replicating domains is linked to mitotic cell division. Nature Genetics, 50(4), 591–602. 10.1038/s41588-018-0073-4 29610480 PMC5893360

