# Supplementary figures and images for: DNA methylation signatures in Blood DNA of Hutchinson–Gilford Progeria syndrome
Source: Aging Cell. 2022 Jan 19;21(2):e13555. doi: 10.1111/acel.13555 (PMC8844112; doi:10.1111/acel.13555)

$-\log_{10}$  binomial p-value

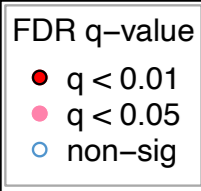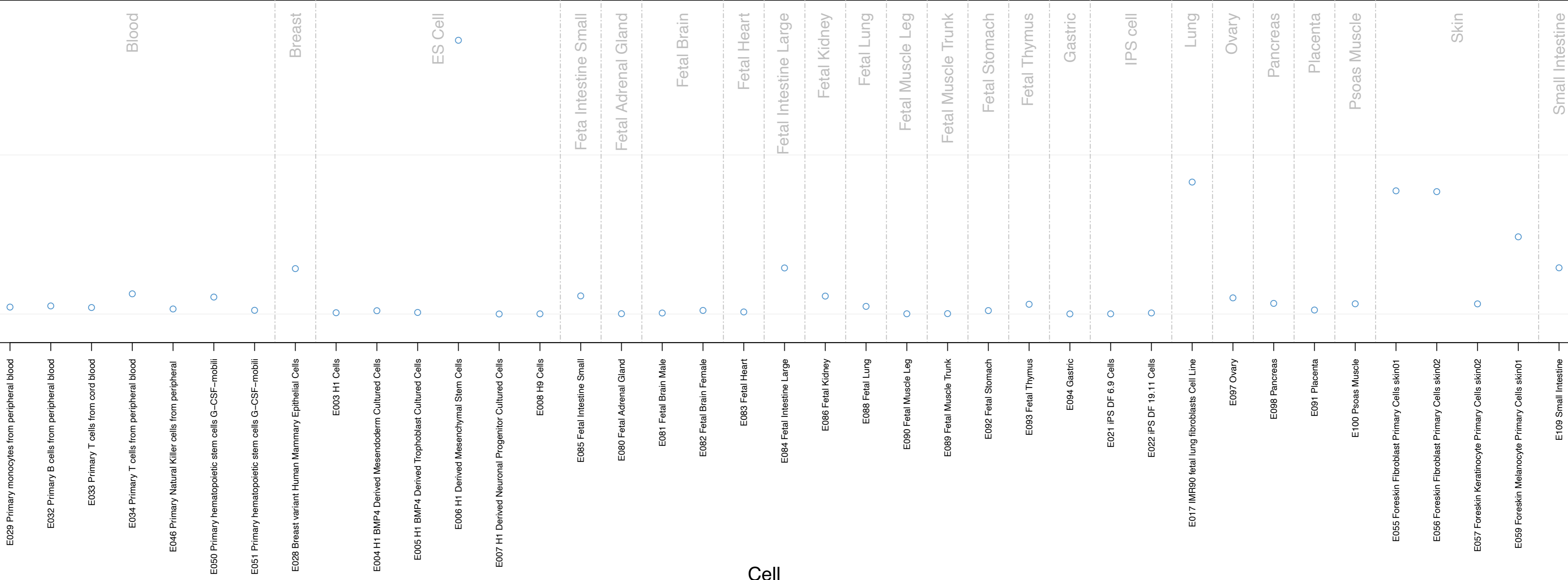

Supplement: Supplementary file 3 — Fig S1 [file ACEL-21-e13555-s002.pdf]

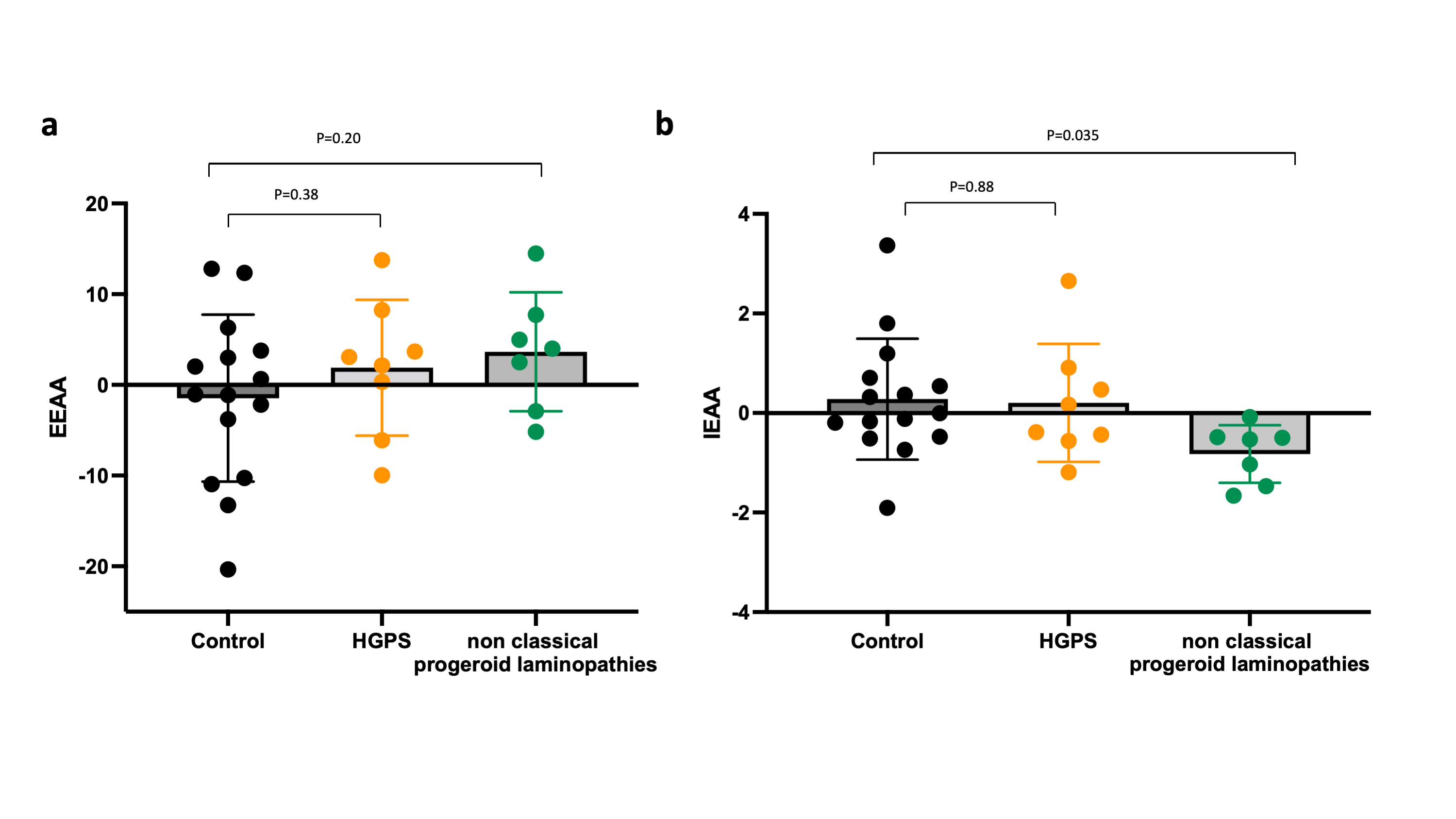

Supplement: Supplementary file 4 — Fig S2 [file ACEL-21-e13555-s006.tiff]

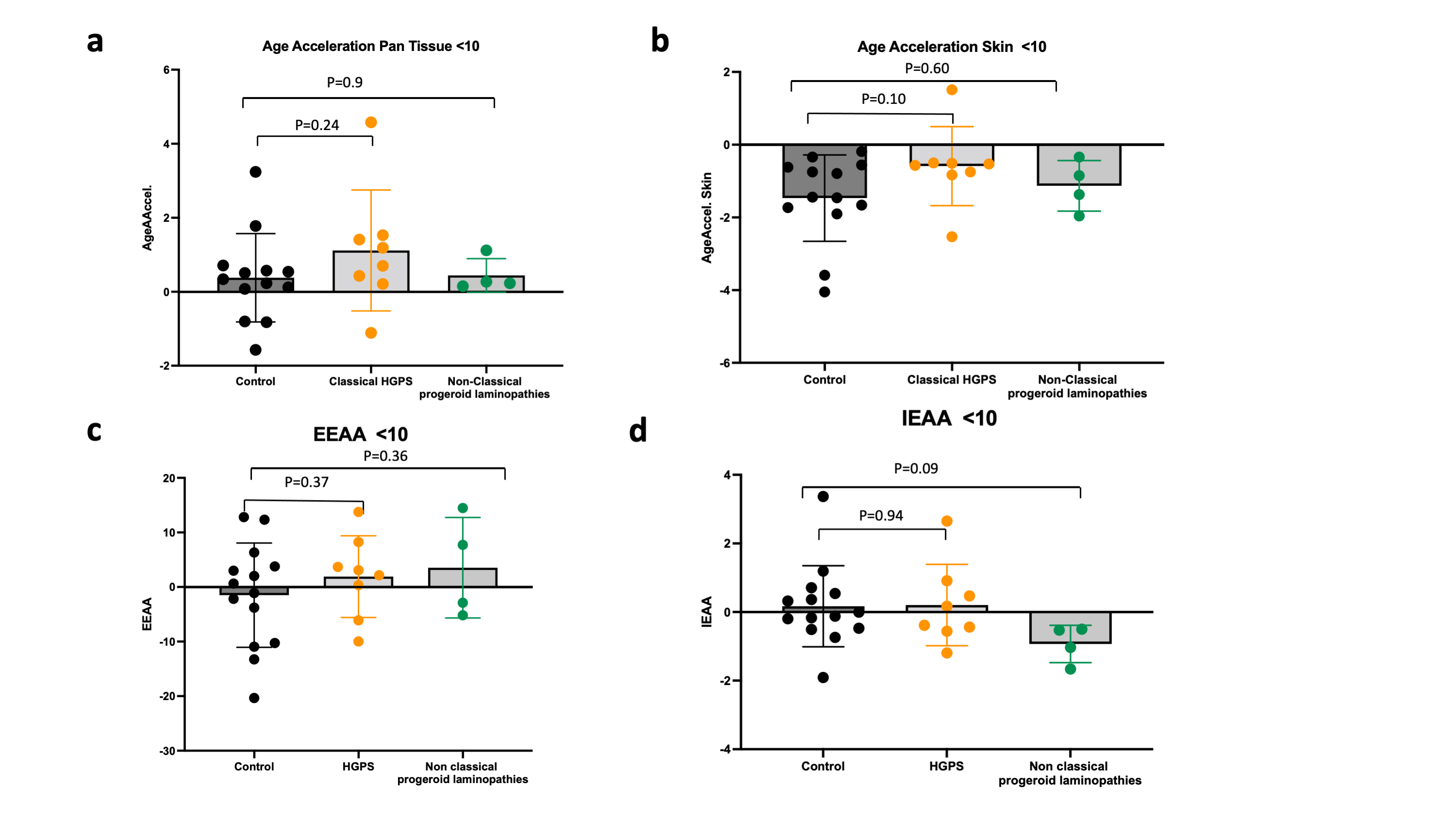

Supplement: Supplementary file 5 — Fig S3 [file ACEL-21-e13555-s001.tiff]

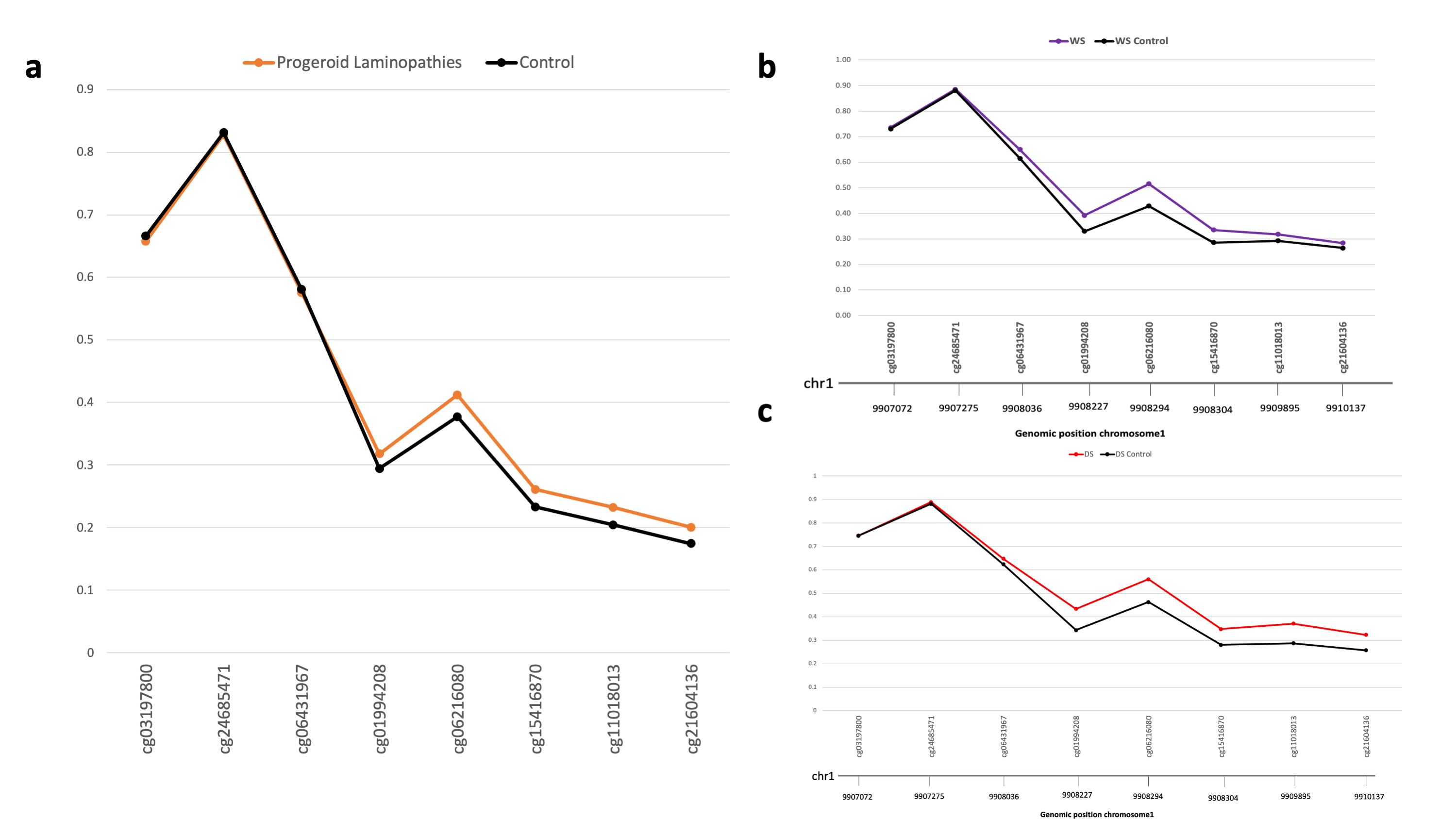

Supplement: Supplementary file 6 — Fig S4 [file ACEL-21-e13555-s005.tiff]
